# Acute SIV Infection in Sooty Mangabey Monkeys Is Characterized by Rapid Virus Clearance from Lymph Nodes and Absence of Productive Infection in Germinal Centers

**DOI:** 10.1371/journal.pone.0057785

**Published:** 2013-03-05

**Authors:** Amanda J. Martinot, Mareike Meythaler, Lu-Ann Pozzi, Karen Dalecki Boisvert, Heather Knight, Dennis Walsh, Susan Westmoreland, Daniel C. Anderson, Amitinder Kaur, Shawn P. O’Neil

**Affiliations:** 1 Division of Comparative Pathology, New England Primate Research Center, Harvard Medical School, Southborough, Massachusetts, United States of America; 2 Division of Immunology, New England Primate Research Center, Harvard Medical School, Southborough, Massachusetts, United States of America; 3 Division of Research Resources, Yerkes National Primate Research Center, Emory University, Atlanta, Georgia, United States of America; 4 Institut für Klinische und Molekulare Virologie Friedrich-Alexander-Universität Erlangen-Nürnberg, Erlangen, Germany; University of Pittsburgh Center for Vaccine Research, United States of America

## Abstract

Lymphoid tissue immunopathology is a characteristic feature of chronic HIV/SIV infection in AIDS-susceptible species, but is absent in SIV-infected natural hosts. To investigate factors contributing to this difference, we compared germinal center development and SIV RNA distribution in peripheral lymph nodes during primary SIV infection of the natural host sooty mangabey and the non-natural host pig-tailed macaque. Although SIV-infected cells were detected in the lymph node of both species at two weeks post infection, they were confined to the lymph node paracortex in immune-competent mangabeys but were seen in both the paracortex and the germinal center of SIV-infected macaques. By six weeks post infection, SIV-infected cells were no longer detected in the lymph node of sooty mangabeys. The difference in localization and rate of disappearance of SIV-infected cells between the two species was associated with trapping of cell-free virus on follicular dendritic cells and higher numbers of germinal center CD4^+^ T lymphocytes in macaques post SIV infection. Our data suggests that fundamental differences in the germinal center microenvironment prevent productive SIV infection within the lymph node germinal centers of natural hosts contributing to sustained immune competency.

## Introduction

Sooty mangabey (SM) monkeys (*Cercocebus atys*) are one of many species of Old World African nonhuman primates that serve as natural reservoir hosts of simian immunodeficiency virus (SIV) infection [1], [2]. In contrast to Asian macaques, SIV-infected natural hosts maintain normal numbers of CD4^+^ T lymphocytes and resist the development of immune deficiency and simian AIDS despite lifelong SIV infection, persistent virus replication and elevated plasma viral loads [3]–[4]. By studying divergent outcomes of SIV infection in natural host species, compared to the highly AIDS-susceptible macaque species, the nonhuman primate model of SIV infection offers unique opportunities to understand HIV pathogenesis. Studies of chronic SIV infection in Indian origin rhesus macaques (RM) have shown that lymphoid tissues undergo similar morphologic changes in architecture throughout the course of SIV infection as seen in chronic HIV infection [5], [6]. Early studies of HIV infection suggest that follicular hyperplasia and persistent immune activation lead to lymphoid follicular “burn out” and the general immune dysfunction seen with the progression to clinical AIDS [7]. Although normal lymphoid architecture is maintained throughout chronic SIV infection in SM [3], [4], [8] little is known about lymphoid changes in acute SM SIV infection.

The germinal center (GC) microenvironment likely plays a pivotal role in HIV pathogenesis dictating disease course and progression in the earliest stages of HIV infection [7], [9], [10] [11], [12] [13]–[14] [15], [16], [17] [18] [19], [20], [21]. FDC have been shown to trap infectious virions within immune complexes (IC), serving as not only a reservoir for latent virus, but also as an archive of HIV quasispecies [17], [20] [21]–[22]. IC have also been shown to prevent shedding of gp120 and to maintain HIV infectivity even in the presence of neutralizing antibody [17] [23], [24]. This, coupled with high levels of TNF-alpha expression in the presence of activated HIV target cells makes the GC microenvironment conducive to HIV replication [11] [25].

Studies of immunodeficiency virus distribution within lymph nodes have shown that RM, like humans, trap lentiviral RNA in the form of IC on FDC [7], [26], [27]; however, IC deposition on FDC has not been reported in natural hosts such as African Green monkeys (AGM) [28] and SM *(data not published).* It is well established that SIV-infected natural hosts do not experience chronic immune activation despite persistent viral replication [2];[29], [30] [31]–[32]. To date, GC development during acute SIV infection in a natural host has not been evaluated longitudinally in parallel with localization of virus production. We hypothesize that mechanisms preventing the accumulation of infectious virus in GC are an important adaptation that contributes to the absence of AIDS progression in SIV infected mangabeys. Here we characterize the GC microenvironment during acute SIV infection in SM, a natural host of SIV, and compare it to that of pig-tailed macaques (PM), *Macaca nemestrina*, and show that rapid viral clearance from the LN paracortex and a lack of infected cells within GC distinguishes non-pathogenic from pathogenic SIV infection.

## Methods

### Tissue Selection

Archived peripheral lymph node (LN) biopsy specimens from six SM and three PM infected with the SM origin viruses SIVsmE041 and SIVsmmFGb, respectively, were investigated. Two of the SIVsmE041-infected SM were subjected to in vivo CD8^+^ lymphocyte depletion prior to SIV infection. The mouse-human chimeric CD8 depleting antibody cM-T807 was administered subcutaneously on the day of SIV inoculation (10 mg/kg) and intravenously thereafter at days 3, 7, and 10 (5 mg/kg) post SIV infection. Biopsy specimens had been immersion-fixed in 10% neutral buffered formalin for 6–18 hours prior to routine processing and paraffin embedding. All nonhuman primate research was conducted in accordance with the guidelines of the Care and Use of Laboratory Animals prepared by the National Research Council. All sooty mangabeys were maintained at the Yerkes National Primate Research Center in accordance with institutional and federal guidelines for animal care approved by the Institutional Animal Care and Use Committee (IACUC) of Emory University under IACUC protocol 226–2003Y. All pig-tailed macaques were maintained at the New England Primate Research Center in accordance with institutional and federal guidelines for animal care approved by the Harvard Medical School IACUC under protocol 03751. Environmental enrichment was provided to all animals. Macaques and mangabeys were monitored daily for evidence of disease including changes in appetite, attitude, or behavior suggestive of illness. Appropriate clinical support was administered under the direction of the attending veterinarian and included analgesics, antibiotics, intravenous fluids, and other supportive care. Macaques were euthanized when they presented with advanced stages of AIDS; criteria for euthanasia included 15% weight loss in two weeks, unresponsive opportunistic infection, persistent anorexia, intractable diarrhea, progressive neurologic signs, significant cardiac or pulmonary signs or other serious illness. In the absence of progression to AIDS, macaques were euthanized per protocol at pre-determined time-points. Macaques were anesthetized with ketamine HCl followed by euthanasia via infusion of sodium pentobarbital to effect by an attending veterinarian. None of the sooty mangabeys were euthanized as part of this study.

### Immunohistochemistry

Immunohistochemistry (IHC) was performed on serial sections of formalin-fixed, paraffin-embedded (FFPE) LN biopsy specimens using standard immunohistochemical techniques [29]. Reactivity for CD20, (clone L26), Ki67 (clone MIB-1), and proliferating cell nuclear antigen (PCNA, clone PC10) were used to delineate germinal centers within B cell follicles (Dako Corp., Carpinteria, CA). T lymphocytes were identified with polyclonal anti-CD3 (DAKO) and monoclonal anti-CD8 and anti-CD4 antibodies (Ab) (clones IA5 and 1F6, respectively, Vector Laboratories, Burlingame, CA). Macrophages were identified with anti-Iba1 (ionized calcium binding adaptor molecule 1,Wako, Richmond, VA). Apoptotic cells were localized with anti-cleaved caspase 3 (Asp175, Cell Signaling Technologies, Danvers, MA). C-C chemokine receptor type 5 (CCR5) expression was evaluated using monoclonal antibody clone 3A9 (BD Pharmingen, Franklin Lakes, NJ).

### In situ Hybridization

Virion-associated RNA and productively infected cells were identified by in situ hybridization (ISH) for SIV RNA in short-fixed paraffin embedded (SFPE) sections as described elsewhere [33].

### Image Analysis

For all IHC and ISH analyses, GCs were imaged using an Optronics DEI-750 CCD camera (Meyer Instruments, Inc., Houston, TX) mounted on an Olympus AH-2 microscope (Olympus America, Inc., Center Valley, PA). GC area was quantified based on Ki67 immunoreactivity, while follicular area was measured as CD20 immunopositivity on serial sections. Total LN area was quantified with Leica QWin software (Leica Microsystems, Inc., Bannockburn, IL) as described elsewhere [33]. SIV-infected cells and caspase 3^+^ cells in the LN paracortex and within GC were quantified manually at 200×magnification. Semi-quantitative scoring of CD4, CD8, and Iba1 immunoreactivity was performed independently by two veterinary pathologists (AJM and SVW) by examining five randomly-selected GC per LN section.

### Confocal Microscopy

Indirect immunofluorescence and confocal microscopy was used to identify and colocalize CD4 and CD3 positive cells within PCNA^+^ germinal centers. Deparaffinized tissue sections were processed using modified IHC protocols [34], followed by the addition of goat anti-mouse IgG2a AlexaFluor 488, goat anti-mouse IgG1 AlexaFluor 568 and goat anti-rabbit IgG AlexaFluor 633 (Molecular Probes, Invitrogen). Two to five representative GCs (identified by the shape and density of PCNA positive cell aggregates) were imaged per section. CD4 and CD3 colocalization within GCs was visualized using a Leica TCS SPI laser-scanning microscope with an upright DMRBE microscope equipped with argon (488 nm), krypton (568 nm), and helium neon (633 nm) lasers. Merged images were processed using Adobe Photoshop CS3 (Adobe Systems Incorporated) with Fovea-Pro image analysis plug-in (Reindeer graphics, Raleigh, NC) to calculate total GC area and area fraction (AF) of fluorescence.

### Statistical Analyses

All analyses were performed using GraphPad Prism version 5 (La Jolla, CA). Differences within groups were analyzed by one-way analysis of variance (ANOVA). Parametric (Student’s t-test) and non-parametric (Mann-Whitney U test, MWU) statistical analyses were used to compare time-points based on the normal distribution. Welsh corrections were applied if variances were unequal. Significant differences were assumed for probability values of p<0.05. Correlation between plasma viral load and tissue virus burden was performed using the Spearman test.

## Results

### Sooty Mangabeys have Accelerated Germinal Center Reactions by 6 Weeks Post-infection Compared to Pig-tailed Macaques

**Figure 1:**
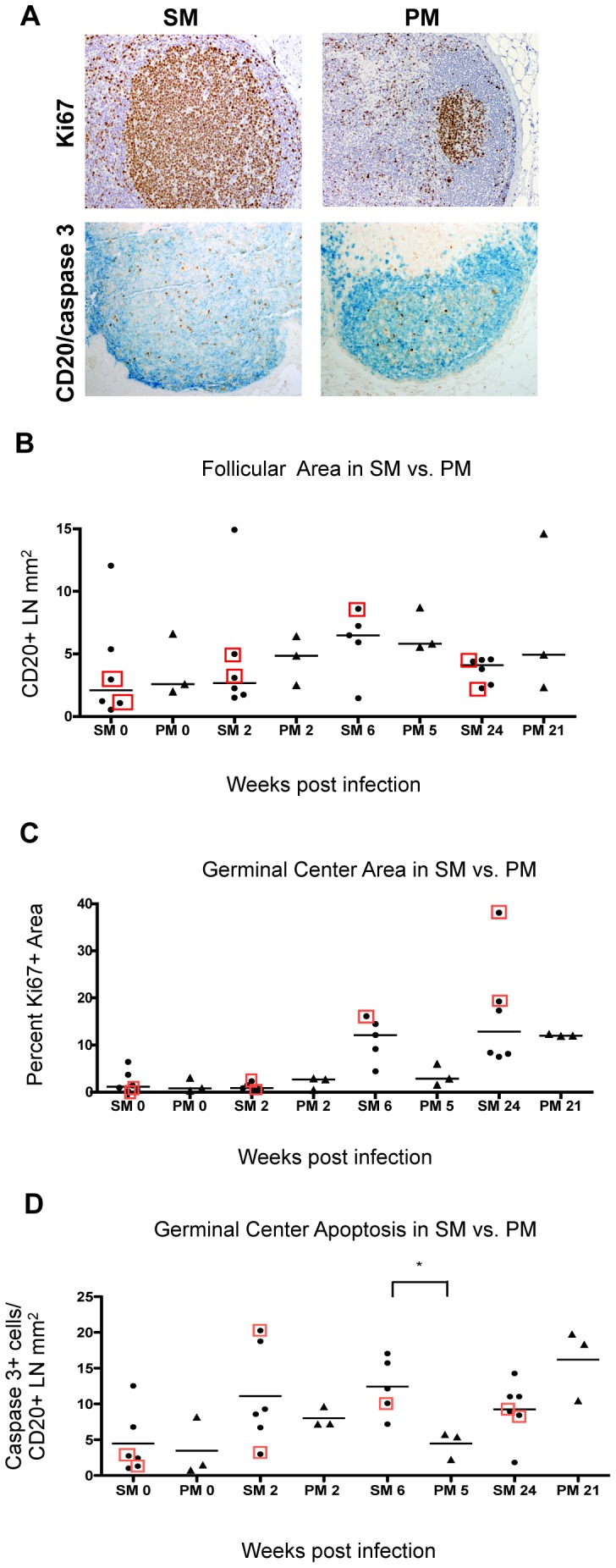
Follicular development and apoptosis within GC. **A**, (**Top**) IHC for Ki67 expression in activated lymphocytes within GC of SM (wk 6) and PM (wk 5). Positive cells indicated by brown 3,3′ diaminobenzadine (DAB) chromogenic substrate; Mayer’s hematoxylin counterstain. **(Bottom)**, Dual IHC for CD20^+^ B lymphocytes to delineate follicles (labeled with Vector Blue chromogenic substrate), and cleaved-caspase 3 to identify cells undergoing programmed cell death (labeled with brown DAB chromogenic substrate). Counterstain was omitted. All photos taken at original magnification **200x. B,** Follicular development is reported as total CD20^+^ area in mm^2^ per biopsy. **C**, Percent germinal center area was quantified as the Ki67^+^ region that included the zones of centroblasts (i.e., dark zones) and activated centrocytes (i.e., light zones) within follicles, divided by the total LN section area. **D,** Apoptosis was measured by number of cells positive for cleaved-caspase 3 (caspase 3^+^) by IHC in B cell areas (CD20^+^). Both SM and PM had progressive germinal center reactions shown by the trend of increasing germinal center size with increasing apoptosis within germinal centers. SM have more apoptotic cells in GC at 6 wpi compared to PM at 5 wpi (p = 0.035). The number of caspase 3^+^ cells decrease in SM by 24 wpi, while the number of apoptotic cells in PM continues to increase (trend). CD8 depleted animals were included in the statistical analysis and are indicated by boxes. Pairs of time points were analyzed (MWU) for difference between medians (bars).

We characterized the germinal center reaction in the first 24 weeks following SIV infection in SM and PM by IHC analysis of serial sections of peripheral LN biopsies with multiple cellular and proliferation markers (Figure 1A–D). SM and PM had comparable follicular (B lymphocyte) and GC area measurements at baseline and at 2 weeks post-inoculation (wpi) with SIV (Figure 1B, C **)**; however, by 6 wpi, SM showed a trend towards greater Ki67^+^ GC area and had significantly greater numbers of apoptotic cells (caspase 3^+^) within GC as compared to PM at a similar time post-inoculation (**Fig. 1C**, p = 0.035). By 21 wpi, PM showed a trend towards higher numbers of apoptotic cells in GC. The difference in kinetics of GC apoptosis (predominantly activated B lymphocytes) suggest that compared to PM, SM germinal center reactions occur more rapidly in response to immune stimulation with SIV. This finding is consistent with what has been reported in acute SIV infection of African Green monkeys [35]. Exclusion of the two CD8-depleted SM from the analysis did not affect trends in GC size, development, or degree of apoptosis among SM (data not shown).

### Rapid Decline in Cell-associated SIV in Peripheral Lymph Nodes of Sooty Mangabeys in Acute SIV Infection

**Figure 2:**
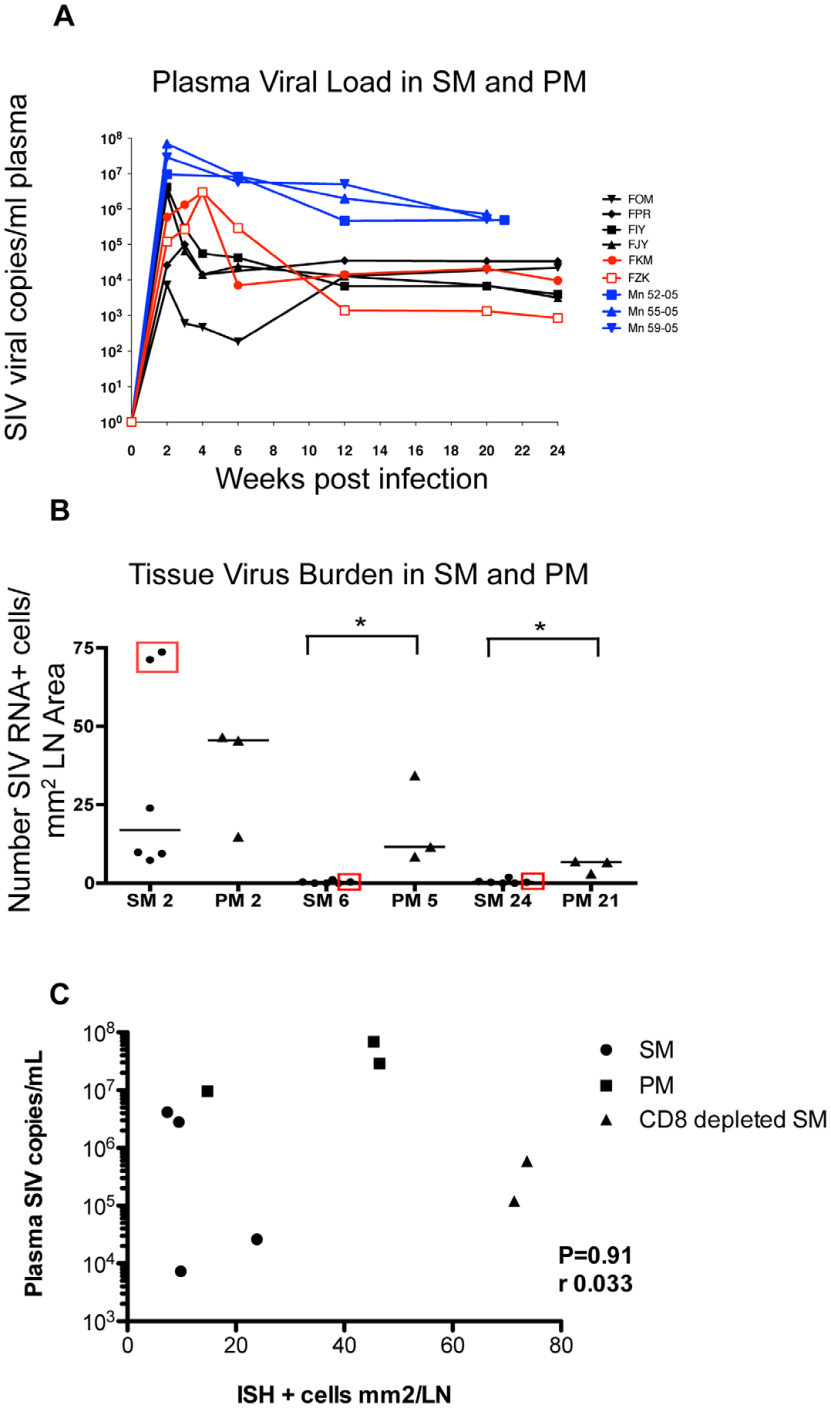
Plasma and tissue viremia in SM and PM during acute SIV infection. **A**, SIV viral copies per ml of plasma longitudinally in CD8-depleted and non-depleted SM versus PM. **B,** SIV infected cells per mm^2^ LN area. Infected cells indicated by RNA in situ hybridization in short-fixed paraffin embedded tissue sections of peripheral lymph node at 2, 6 and 24 wpi in SM and 2, 5 and 21 wpi in PM. Number of infected cells was quantified as NBT/BCIP positive cells per mm^2^ lymph node section area. PM have higher median number of infected cells per mm^2^ at 5 wpi (p = 0.03), and 21 wpi (p = 0.03). Differences in medians were analyzed by MWU. Median virus burden was not significantly different at 2 wpi between SM and PM. SIV+ cell counts for CD8-depleted SM are indicated in boxes. **C,** Correlation between plasma SIV viral copies at 2 wpi and LN virus burden as measured by number of ISH^+^ cells per mm^2^ LN. No correlation is seen, Spearman’s correlation coefficient (r) = 0.033, p = 0.91.

Plasma SIV RNA levels at two weeks post SIV infection ranged between 7.3×10^3^ and 4×10^6^ copies/ml in SM and between 9.5×10^6^ and 6.9×10^7^ copies/ml in PM (Figure 2A). At this time-point, the cell-associated SIV burden in the LN was comparable in SM and PM (Figure 2B). Thus, the higher viremia levels at 2 wpi in the PM compared to SM did not correlate with the cell-associated SIV burden in the LN as evaluated by SIV RNA ISH (Figure 2B). In fact, no correlation was noted between plasma viral load and peripheral LN tissue viral burden as quantified by ISH (p = 0.91, Spearman r = 0.033; Fig. 2C). The highest cell-associated tissue virus burden among SM and PM was observed in LN from the two CD8-depleted SM, even though viremia at 2 wpi for these animals was at least one-log lower than that observed for PM (Figure 2A–B). When the two CD8-depleted SM were excluded from the analysis, SM showed a trend towards lower overall LN virus burden at 2 wpi compared with PM, but this difference did not reach statistical significance (p = 0.11, MWU; data not shown). From 6 wpi onwards, infected cells were rarely detected in the LN of SM, including the CD8-depleted SM, while numerous infected cells continued to be detected in peripheral LN of PM (Table 1, Fig. 2B). The decline in SIV-infected cells in the LN of the CD8-depleted SM coincided with recovery of CD8^+^ T lymphocytes (data not shown). SM had significantly lower LN virus burden at 6 wpi compared to PM at 5 wpi (p = 0.03) and at 24 wpi compared to PM at 21 wpi (p = 0.03) (Figure 2B). A similar difference in post-acute SIV infection LN viral burden and discordance with plasma virus level was noted between SM and SIVmac239-infected rhesus macaques [36]. These data suggest faster clearance of tissue-associated SIV-infected cells in SM, or alternatively, may reflect differences in target cell availability in the LN of mangabey versus macaque species.

**Table 1:** Localization of SIV infected cells in LN from SM and PM.

	SIV Productively Infected Cells					
	Paracortex			Germinal Center		
	SM	CD8-depleted SM	PM	SM	CD8-depleted SM	PM
**Wk2**	+++	++++	+++	− (n = 24)	+* (n = 15)	++ (n = 42)
**Wk 5/6**	+	+	+++	− (n = 80)	+* (n = 43)	+++ (n = 56)
**Wk21/24**	+	+	++	− (n = 54)	+* (n = 23)	++ (n = 80)

Infected cells, as quantified by ISH for SIV RNA. Histomorphological localization of infected cells (identified by dark blue NBT/BCIP chromogen) in GC versus paracortical microenvironments was accomplished by microscopic analysis of Nuclear Fast Red counterstain. Scoring is based on the average for each group at each time point, divided into quartiles (+; 0–5/mm^2^) (++; 6–15/mm^2^); (+++; 16–50/mm^2^); (++++; >50/mm^2^) derived from quantitative measurements. Asterisk (*) Indicates one germinal center contained an SIV-infected cell at 2, 6, and 24 wpi in a CD8 depleted SM. Numbers of GC evaluated at each time-point is indicated by n values.

### Differential Localization of SIV-infected Cells in the Lymph Nodes of Sooty Mangabeys Versus Pig-tailed Macaques during Acute SIV Infection

**Figure 3:**
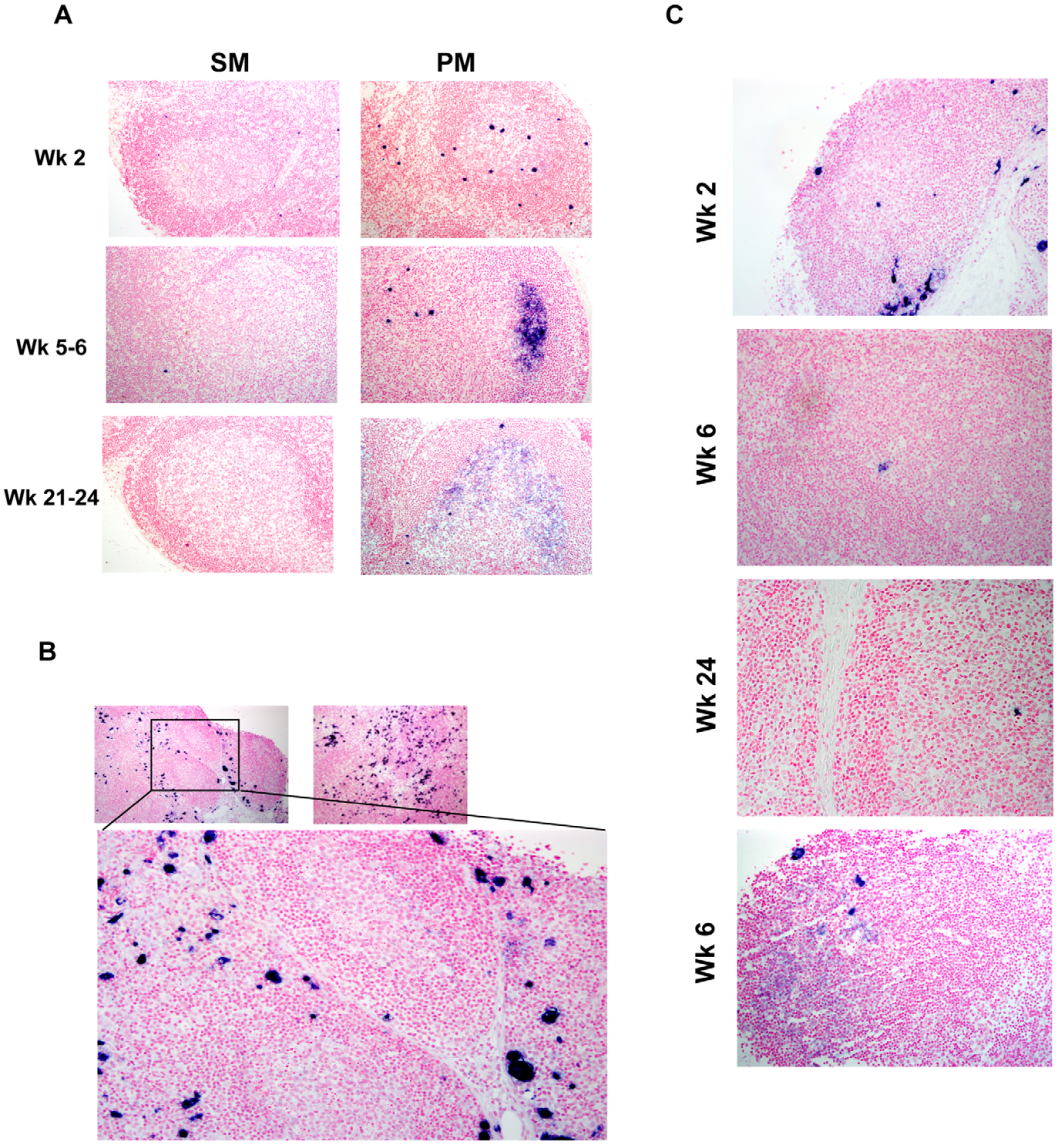
ISH for SIV RNA in peripheral LN from SM and PM. ISH was performed using short-fixed paraffin embedded tissue sections of peripheral lymph node collected at 2, 6 and 24 wpi in SM and 2, 5 and 21 wpi in PM. **A,** SIV-infected cells were not observed in GC of immune competent SM but were readily apparent in GC of PM at all time points. Diffuse hybridization signal characteristic for the presence of immune-complexed virions trapped by FDC was not observed in SM, but was identified in PM at all time points, with the most significant trapping seen at 5 and 21 wpi. **B,** Despite higher tissue viral burden in CD8 depleted SM compared to PM, germinal centers typically lack infected cells and virus trapping despite close proximity to numerous infected cells in the lymph node paracortex. **C,** GC in CD8-depleted SM at 2, 6 and 24 wpi. Only one infected cell was localized within a GC from a SM at any given time point in CD8-depleted SM. Virus trapping was also extremely rare in CD8 depleted SM (bottom).

In addition to the total LN viral burden, we also evaluated the quantity and pattern of distribution of SIV-infected cells in the LN of the two species. SIV RNA hybridization signal at two weeks post SIV infection revealed numerous infected cells in the LN paracortex of both species (Table 1). However, the GC areas showed a striking species-specific discordance with only the PM showing consistent presence of infected cells within developing GC at all time-points (Table 1, and Figure 3A). Infected cells were not detected in the GC of the four non-CD8-depleted SM at any time-point in the first 24 weeks post SIV infection (Table 1 and Figure 3A), and only rare infected cells (one positive cell per animal per time point) were observed in the GC of the two CD8-depleted SM at 2, 6, and 24 wpi, (Table 1 and Figure 3C). It is striking that CD8-depleted SM showed only rare infected cells in the GC areas despite having cell-associated LN virus burdens at 2 wpi that exceeded those present in all other animals including the PM (Figure 2–3B, Table 1). Moreover, all three PM showed evidence of infected cells within the GC even though the total LN virus burden in one PM was similar to the non-CD8-depleted SM (Figure 2B). These data complement the recent report by Brenchley *et al.* showing decreased numbers of infected cells in germinal centers of SM during chronic SIV infection and confirms that differential localization is a feature that distinguishes pathogenic from non-pathogenic SIV infection early during the course of SIV infection [37].

Along with the absence of productively-infected cells, the GC of immune competent SM also lacked evidence of the diffuse SIV hybridization signal which indicates trapping of immune-complexed virions by FDC in the GC light zone (zone of centrocytes); in contrast, PM exhibited robust diffuse ISH signal within GC consistent with virus trapping (Figure 3A). In one of the two CD8-depleted SM, a single, focal region of diffuse hybridization (suggestive of virus trapping) was observed; however, the microanatomy of this site could not definitively be identified as a GC (Figure 3C bottom panel).

### Sooty Mangabeys have Fewer CD4^+^ SIV Target Cells within Germinal Centers Compared to Pig-tailed Macaques

**Figure 4:**
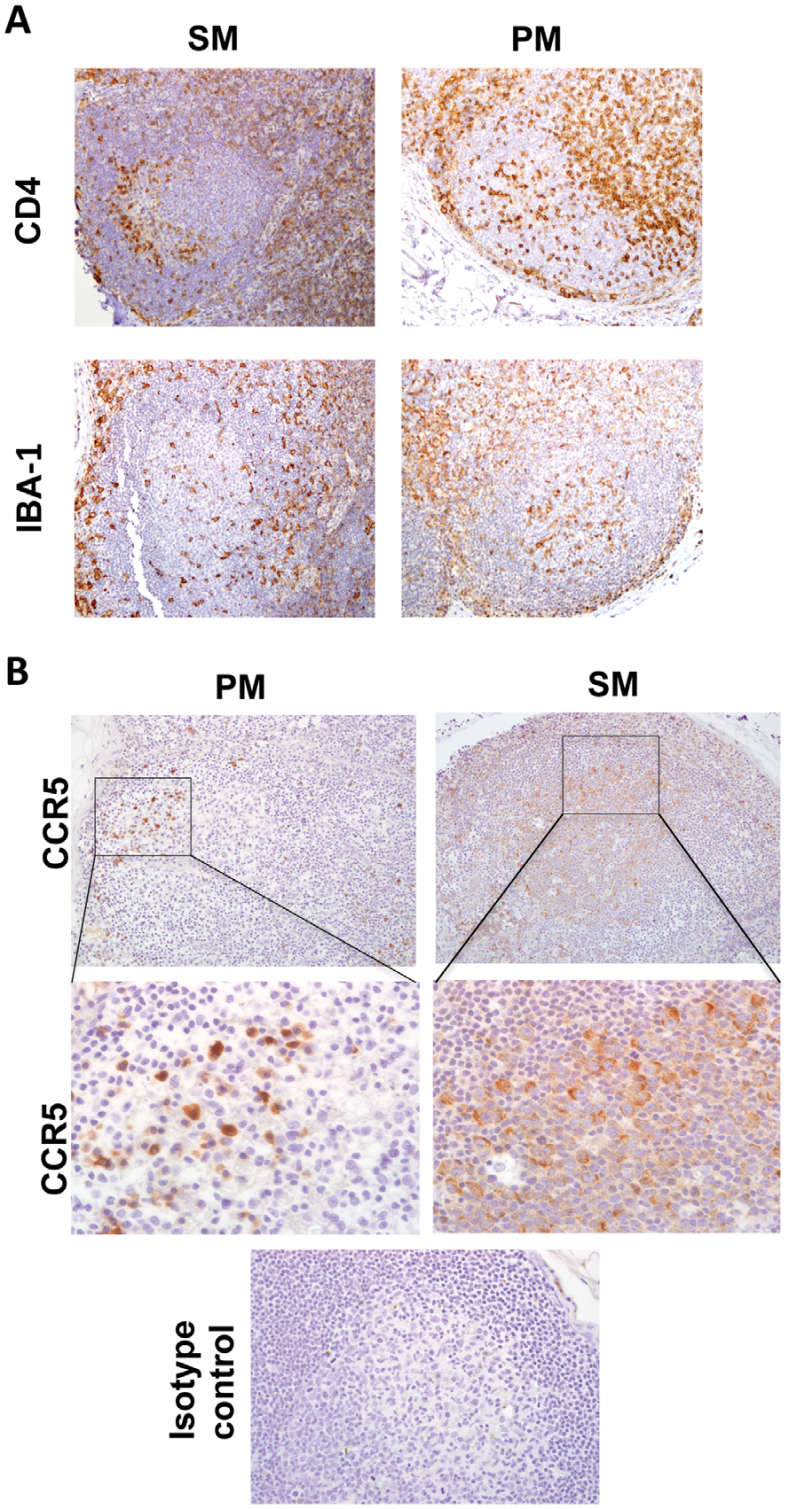
Immunophenotype of resident GC cells in SM and PM by IHC. A, (Top) CD4 IHC at 2 wpi in SM and PM. **(Bottom),** Iba-1 IHC at 2 wpi in SM and PM, All images are original magnification 200x. **B,** (**Top**) CCR5 IHC at 6 wpi in SM and PM. Original magnification 200x **(Middle),** CCR5 positive cells within germinal center, **(Bottom)** isotype negative control. Original magnification 600x. Immunoreactive cells in all images are labeled with brown DAB chromogen; blue hematoxylin counterstain reveals tissue morphology. All photos are representative examples.

**Figure 5:**
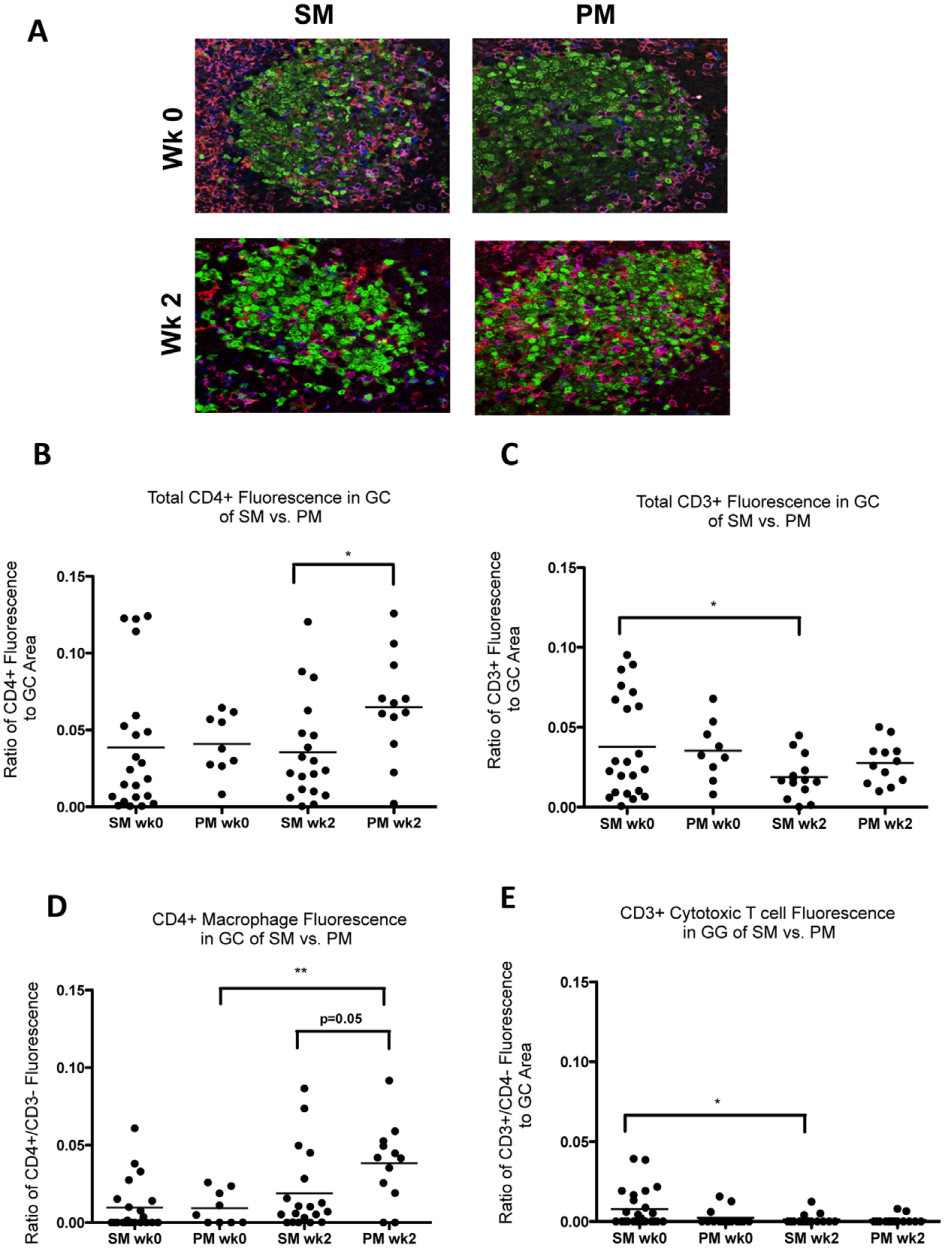
Immunophenotype of resident GC cells by confocal microscopy. A, Localization of CD3^+^ and CD4^+^ resident cells in germinal centers. Multiparameter confocal microscopy was used to identify CD3^+^ and CD4^+^ cells within germinal centers (PCNA^+^) of lymph nodes from SM and PM at baseline and 2 wpi by simultaneous localization of CD3 with Alexafluor 633 (blue), CD4 with Alexafluor 568 (red) and PCNA with Alexafluor 488 (green). CD4^+^ T lymphocytes (CD3^+^/CD4^+^) were identified by purple/pink co-localization of Alexafluors 633 (blue) and 568 (red). Macrophages (CD3^−/^CD4^+^) were identified by localization of red Alexafluor 568. By default, CD8^+^ T lymphocytes (CD3^+^/CD4) were identified by localization of Alexafluor 633 (blue). Five randomly selected GC were imaged for most animals; however, all available GC were imaged when fewer than 5 GC were present per section. Magnitude of fluorescence (panels B through E) is reported as ratio of area fraction (AF) of fluorescent pixels within GC to area fraction of PCNA^+^ (total germinal center) fluorescent pixels, as calculated using an algorithm in the imaging software (Fovea Pro). Each point represents a germinal center, with horizontal bars indicating the mean. **B,** PM had greater numbers of CD4^+^ cells in GC at 2 wpi than SM (p = 0.02). **C,** No difference was noted in the mean number of CD3^+^ cells in GC between SM and PM at baseline and 2 wpi. However, numbers of CD3^+^ cells were higher at baseline than at 2 wpi in SM (p = 0.02). Values for CD8-depleted SM were excluded from the 2 wpi data set. **D,** CD3**^−/^**CD4^+^ fluorescence (macrophages), calculated by subtracting CD3^+^ AF from the total CD4^+^ AF. Negative ratios reported as zero. PM had higher numbers of GC macrophages at 2 wpi compared to SM (p = 0.05). Mean number of GC macrophages increased in PM from baseline to 2 wpi (p = 0.003). **E,** CD3^+^/CD4^−^ fluorescence calculated by subtracting total CD4^+^ AF from CD3^+^ AF. Negative ratios reported as zero. Low numbers of CD3^+^/CD4^−^ cells (likely cytotoxic CD8^+^ T lymphocytes) were observed in GC for both species at all time points. SM have higher baseline numbers of (CD8^+^ cells) compared to 2 wpi (p = 0.02). Differences in mean area fractions between time points were analyzed using unpaired Student’s t-tests. Welsh’s correction was used if variances were unequal. Values for CD8-depleted SM were excluded from the2 wpi data set.

The discordant localization patterns of SIV-infected cells in the LN of SM and PM which could not be attributed to local or systemic viral loads prompted us to further characterize the GC microenvironment in both species to determine whether other factors, such as target cell localization, might explain the differences observed between the two species during acute SIV infection. The pattern of viral decay in the plasma of SM following onset of potent antiretroviral therapy suggests that short-lived activated CD3^+^/CD4^+^ T lymphocytes are the primary target cells for SIV replication in both SM and RM [38]. To determine whether differences in relative numbers of target cells existed within GC of SM versus PM during acute infection, the phenotype and relative number of immune cells within GC were characterized via IHC. LN biopsy sections were incubated with anti-CD4, anti-CD8, anti-Iba1 and anti-CCR5 antibodies. Semi-quantitative scoring was performed for CD4, CD8, and Iba-1 immunoreactivity (Table 2 and Fig. 4). Species-specific differences were not apparent at baseline for CD4^+^, CD8^+^, and CCR5^+^ cells in the GC. Species-specific differences in antibody affinity for CCR5 precluded semi-quantitative scoring of CCR5 reactivity; however, CCR5 positive cells were detected in the GC of both PM and SM and most likely represent a combination of CCR5+ B lymphocytes, mononuclear cells and fewer numbers of T lymphocytes (Figure 4B). PM had slightly greater numbers of CD4^+^ T lymphocytes at 2, 6, and 24 wpi within GC compared to SM at similar time points (Table 2 and Figure 4A). PM had slightly greater numbers of Iba1 positive macrophages at baseline and at 2 wpi than SM; however, numbers of GC macrophages increased dramatically in both species at later time points (Table 2). Early studies revealed a correlation between the presence of HIV-infected cells in GC and increased numbers of CD8^+^ cytotoxic T lymphocytes (CTL) within human LN follicles [6], [9], [39], and recent reports suggest that SIV-specific CTL likely contribute to viral clearance in SM LN as well [36]. Indeed, PM had slightly increased numbers of CD8^+^ T lymphocytes in GC at 6 and 21 wpi (Table 2), correlating temporally with the greatest abundance of cell-associated and cell-free virus within GC; however, the antigen specificity of the CD8^+^ cells was not determined and the overall number of CD8^+^ T lymphocytes in GC was very low in both species. Given that SM cleared most SIV-infected cells from the LN by 6 wpi, we re-evaluated GC at 0 and 2 wpi via confocal microscopy in an effort to better characterize the phenotype of resident GC immune cells that could serve as targets for SIV replication during acute infection. SFPE sections of LN were triple-labeled with anti-human CD4, CD3, and PCNA antibodies, followed by fluorochrome-conjugated secondary antibodies (Fig. 5A). SM and PM had similar numbers of CD3^+^ lymphocytes in GC at 0 and 2 wpi (Fig. 5C); however, SM had significantly fewer CD3^+^/CD4^+^ cells (CD4^+^ T lymphocytes) in GC at 2 wpi compared to PM (Figure 5B; p = 0.02) consistent with semi-quantitative scoring of IHC assays. Indirect measurement of CD3^−/^CD4^+^ fluorescence suggested a trend towards lower numbers of macrophages at 2 wpi in SM compared to PM (Figure 5D; p = 0.05). A significant increase in CD3^−/^CD4^+^ fluorescence was seen in GC of PM but not SM from 0 to 2 wpi (Fig. 5D, p = 0.003). CD3^+^/CD4^−^ fluorescence (likely CD8+ cytotoxic T lymphocytes) was low at all time-points, consistent with IHC analysis (Fig. 5E and data not shown), although SM had higher baseline numbers compared with SM at 2 wpi (p = 0.02). These findings helped clarify the phenotype of the CD4^+^ cells in PM seen with IHC at 2 wpi and suggest that numbers of SIV target cells such as T follicular helper cells (T_FH_) and macrophages are increased in the GC of PM while remaining constant in SM during the earliest stages of SIV infection.

**Table 2:** Immunohistochemistry for CD4, CD8, and IBA-1 in GC of SM and PM.

CD4+ IHC Score in Germinal Center		
	SM	PM
Wk 0	+	+
Wk 2	++	+++*
Wk 6/5	++	+++
Wk 24/21	+++	++++
**IBA-1+ IHC Score in Germinal Center**		
	**SM**	**PM**
Wk 0	++	+++
Wk 2	++	+++*
Wk 6/5	++++	++++
Wk 24/21	++++	+++++
**CD8+ IHC Score in Germinal Center**		
	**SM**	**PM**
Wk 0	+	+
Wk 2	+	+
Wk 6/5	+	++
Wk 24/21	+	++

* indicate accompanying image in Fig. 4.

Mean score for DAB staining at each time point post-SIV inoculation. PM have higher CD4 scores at 2, 5, and 21 wpi compared to SM at similar time-points. PM also had slightly increased numbers of IBA-1^+^ cells (macrophages) at 2 wpi compared to SM. CD8 scores are slightly higher for PM at 5 and 21 wpi. Overall number of CD8^+^ cells in GC of both species is extremely low. Asterisks indicate accompanying image in Figure 4A.

## Discussion

During the course of progressive HIV and SIV infection, viral RNA can be localized within two compartments of the GC microenvironment of secondary lymphoid tissues: within productively-infected cells and as cell-free, immune-complexed virions bound to Fc receptors on FDC [20], [40], [41], [42], [43]. GC in secondary lymphoid tissues from HIV infected individuals routinely contain immune-complexed virions [40]; moreover, the presence of infected cells within GC of HIV+ individuals is frequently cited as evidence that GC serve as a reservoir of infectious virus [7], [21], [40]. Evidence suggests that infectious virus particles sequestered within IC as well as virus produced by infected cells within GCs provide a source for new infections of activated CD4^+^ T lymphocytes and macrophages and facilitate ongoing CD4^+^ T cell loss, immune activation, and follicular dissolution. In this longitudinal study of the GC microenvironments in a natural host SM versus non-adapted PM, we have observed significant differences in the pattern of SIV distribution and in the efficiency with which virus is cleared from secondary lymphoid tissues during acute infection. During the acute phase of pathogenic SIV infection, the GC of PM accumulate cell-free SIV particles in light zones (i.e., “FDC trapping”) and contain numerous productively-infected cells, which can still be found in significant numbers at 24 wpi. In contrast, although SIV positive cells are abundant in LN paracortical zones, neither cell-free virus nor productively infected cells are localized within GC during acute, apathogenic SIV infection of SM, and productively infected cells are no longer detectable in lymphoid tissue by 6 wpi. These observations suggest significant divergence between the GC microenvironments of SM versus PM. Although previous studies have noted that SIV infected natural hosts including African green monkeys (AGM) [44] and SM do not trap significant quantities of virus in GC, to our knowledge this is the first study which additionally describes discrete differences in the patterns of cell-associated SIV in GC and documents these disparities through longitudinal analysis of LN from SM in parallel with macaques infected with SM origin viruses. Furthermore, it has recently been reported that this disparity in localization of LN virus is also present in chronically infected SM and HIV positive humans suggesting that this difference in anatomic distribution is stable throughout the course of HIV/SIV infection [37]. We hypothesize that a lack of cell-free virus trapping on FDC in combination with decreased numbers of SIV target cells within GC of SM contributes to lower numbers of infected cells within GC.

With regards to the demonstration of cell-free SIV RNA by ISH, the sensitivity of the assay used in the current study might be a source of error, as all LN biopsy specimens were fixed overnight in 10% formalin, rather than for 4–6 hours, which is optimal for demonstration of cell-free viral RNA. Under these conditions, the lower plasma virus loads in SM relative to PM may have placed the quantity of immune-complexed SIV in SM below the level of detection by ISH. Nevertheless, cell-associated viral RNA can be demonstrated without loss of sensitivity in specimens even after several days of formalin fixation, as evidenced by robust demonstration of productively infected cells in both species. Given, that we found no correlation between plasma viral load and LN virus burden (Fig. 2C) it is unlikely that limited ISH assay sensitivity accounts for the discordance between infected cells in GC between the two species. This further highlights the importance of evaluating plasma viremia in conjunction with tissue specific SIV burden and localization when evaluating differences in SIV immunopathology.

In this and previous studies we have shown that CD8^+^ cells are important for reducing virus burden in secondary lymphoid tissues and plasma in SM, similar to what has been shown for Asian macaques (Kaur *unpublished),* [29], [36], [44]. SM efficiently clear infectious virus from lymphoid tissues by 6 wpi; moreover, CD8^+^ T lymphocytes are central to the control of tissue virus burden as shown by prolific virus replication in the absence of circulating CD8^+^ T cells and rapid reduction of SIV^+^ cells from LN upon reconstitution of CD8^+^ cells in depleted SM (data not shown). The absence of SIV from SM follicular centers in the face of CD8-depletion despite cell-associated lymphoid virus burdens that far exceeded those recorded in PM prompted us to investigate features of the GC microenvironment that might contribute to this divergent phenotype. Relative to PM, SM had fewer CD4^+^ SIV target cells in GC at all acute time-points post-SIV infection and fewer follicular macrophages prior to SIV infection and at 2 wpi. These observations suggest that SM may preserve follicular center function by limiting target cell presence within GC and thereby reducing follicular virus burden. Small cohort size and limitation in LN samples precluded the use of flow cytometric phenotyping of infected cells in this study, but a recent study by Brenchley *et al.* elegantly demonstrated that SM have decreased numbers of SIV infected T_FH_ and central memory T lymphocytes in LN during chronic infection supporting our hypothesis that the increased numbers of CD4+ cells in GC detected by confocal microscopy in this study most likely represent T_FH_ [37].

IHC revealed abundant CCR5^+^ cells in GC on all sections from both species. Given the role of the germinal center reaction on the development of T_FH_ cells and central memory lymphocytes [45] further elucidation of CCR5 expression would be informative in light of recent data by Paiardini *et al.* showing that CCR5 down-regulation prevents SIV infection of central memory T lymphocytes *in vitro* [46]. However, the strong CCR5 expression reported here in GC from both species does not support this explanation for the absence of infected cells within GC of SM. Furthermore Brenchley *et. al.* showed no difference in CCR5 expression between SM and RM on T_FH_ [37]_._ It would also be interesting to understand whether differences in the cellular composition of follicles reflects species specific variations in the cytokine milieu within the GC microenvironment that could impact cell recruitment to the GC.

We hypothesize that restrictions in target cell availability within follicular centers limits the quantity of infectious virus that is produced and retained within the GC of SM, and reduces the risk of infection of resident GC immune cells such as CD4^+^ follicular helper T cells, which are critical for the development of immunologic memory [14], [47], [48], [49], [50], [51], macrophages, and other cells that traffic through the GC. The restriction of productively infected cells from the GC microenvironment may protect follicular centers of SM from lesions associated with the presence of infected cells, including the influx of cytotoxic CD8^+^ T lymphocytes, increased levels of immune activation, and apoptosis [39], [52], [53]. Regardless of the mechanism, we hypothesize that fundamental differences in the anatomic disposition of viral replication in secondary lymphoid tissues significantly contributes to the difference in pathophysiologic outcome of SIV infection in SM versus PM and may shed light on evolutionary adaptations that protect natural reservoir species from the consequences of primate lentivirus infection.
